# Isolation and characterization of novel microsatellite loci for the Eastern Pacific marine sponge *Mycale cecilia* by Illumina MiSeq sequencing

**DOI:** 10.1007/s11033-023-08320-9

**Published:** 2023-04-08

**Authors:** Misha Yazmín Hernández-Lozano, José Antonio Cruz-Barraza, Axayácatl Rocha-Olivares

**Affiliations:** 1 Posgrado en Ciencias del Mar y Limnología, UNAM, Mazatlán, México; 2 Instituto de Ciencias del Mar y Limnología, Universidad Nacional Autónoma de México, Unidad Académica de Mazatlán, Av. Joel Montes Camarena s/n, Mazatlán, Sinaloa, 82000 CP México; 3 Departamento de Oceanografía Biológica, Centro de Investigación Científica y de Educación Superior de Ensenada (CICESE), Ensenada, Baja California México

**Keywords:** Porifera, Mycalidae, Mexican Pacific, Hypervariable markers, Population genetics

## Abstract

**Background:**

*Mycale cecilia* is an abundant Eastern Tropical Pacific sponge living in a wide variety of habitats, including coral reefs where it may directly interact with corals. It is also known to possess secondary metabolites of pharmacological value. These aspects highlight the importance of having a better understanding of its biology, and genetic and population diversity.

**Methods and results:**

In the present study, we isolated and characterized twelve novel microsatellite loci by Illumina MiSeq sequencing. The loci were tested in 30 specimens collected from two coral reef localities (La Paz, Baja California Sur and Isabel Island, Nayarit) from the Mexican Pacific using M13(-21) labeling. All loci were polymorphic, with two to nine alleles per locus. Expected heterozygosities varied from 0.616 to 0.901. Eleven loci were tested and successfully amplified in *M. microsigmatosa* from the Gulf of Mexico.

**Conclusion:**

Here we report the first microsatellite loci developed for a sponge species from the Eastern Pacific coast. These molecular markers will be used for population genetic studies of *M. cecilia*, and potentially in other congeneric species; particularly in vulnerable marine areas that require protection, such as coral reefs.

## Introduction

*Mycale cecilia* is a widespread shallow-water sponge distributed along the tropical Eastern Pacific from Mexico to the Galapagos Islands, with some records in Hawaii [1, 2]. The species typically inhabits a broad variety of habitats, including rocky substrates from bays and estuaries and coral reefs ecosystems, where it may directly interact with corals by covering their branches [3]. This species is also well known for synthesizing secondary metabolites such as mycalazals, mycalenitriles, and related pyrrole-2-carbaldehyde derivatives, which have shown activity as growth inhibitors of various tumor cell lines, in particular against the human prostate cancer cell line [4].

In the last decades, a variety of molecular markers (e.g. sequences from COI mtDNA and 18S, 28S ITS’s nrDNA genes) have been used to establish phylogenetic relationships in sponges [5], and in some cases, to elucidate the phylogeography and genetic connectivity of their populations [6]. However, these markers have shown a low level of intraspecific variability [6, 7], which hindered its application for population studies, highlighting the need to include more variable markers. Novel methodological developments in massive parallel DNA next-generation sequencing technologies allow targeting large fractions of the genome to identify multiple Single Nucleotide Polymorphisms (SNPs) [8]. However, the microbial genetic pool is difficult to separate from the sponge DNA after DNA extraction; hence, identifying sponges’ SNPs from DNA mixed (holobionts and sponges) may be challenging using next-generation sequence data [8].

Microsatellite loci have been shown to be a powerful tool and a good option for populations genetic studies in sponges. Nevertheless, their implementation has been limited to a few studies in the Caribbean, the Atlantic-Mediterranean, Antarctica and the Indo-Pacific regions [e.g., 9, 10, 11, 12]. No microsatellites have yet been developed for poriferans from the Eastern Pacific. Here, we report the isolation of 12 microsatellite markers in *M. cecilia* and preliminary data on their allelic variation in two coral reef localities of the Mexican Pacific coast. In addition, we tested the cross-amplification of these microsatellites in a closely related congeneric species *Mycale microsigmatosa* (from the Gulf of Mexico).

## Materials and methods

### Sample collection, DNA extraction and next-generation sequencing

A specimen of *M. cecilia* was collected from Mazatlán, Sinaloa, México (23°11’09.18” N, 106°25’23.47” W) in October 2015. Genomic DNA was obtained from fresh tissue using Wizard® SV Genomic DNA Purification Kit (Promega, Madison, WI). For next-generation sequencing a genomic DNA library was assembled with the Kapa gDNA library kit (Kapa Biosystems, Wilmington, MA), applying a multiplex index. This library was then sequenced using 1/7 of a single lane (2 × 125 base pairs) in a MiSeq platform (Ilumina, San Diego, CA).

For the evaluation of the novel microsatellite loci, 30 individuals of *M. cecilia* were collected by SCUBA diving from two coral reef areas, 15 from Isabel Island, Nayarit (21°50’31” N, 105°52’51” W) in October of 2018, and 15 from La Paz, Baja California Sur (24°09’31” N, 110°20’26” W) in March 2019. Additionally, eight specimens of the congeneric species *Mycale microsigmatosa* were collected in Laguna de Términos, Campeche in March 2019 (18°38’18” N, 91°47’55” W) and included to tests the cross-amplification of the new microsatellites. For these samples, DNA was extracted using the cetyltrimethyl ammonium bromide (CTAB) protocol [13].

### Design of microsatellite primers and genotyping

All DNA reads were analyzed for quality control using FastQC v.0.10.1 (Babraham Institute, Cambridge, UK) [14], then they were trimmed and subject to *de novo* assembly into contigs with CLC Genomics Workbench v.7.0.3 (CLC bio, Boston, MA) [15]. The search for repetitive motifs for microsatellites (di-, tri-, and tetra nucleotide repeats) and the PCR- primer design were made from the resulting contigs (under the parameters: minimum of 5x coverage, a product size of 110–250 bp and primer length range 19–39 bp) using Msatcommander [16]. The resulting oligos were then synthesized in Macrogen, South Korea. Initially, all primer sets were tested in 10 specimens at annealing temperatures ranging from 54 to 60 °C and through MgCl_2_ concentration gradients. A fluorochrome was incorporated into the complementary universal tail M13(-21) of the forward primer [17], however, amplifications with fluorochrome were only achieved for 16 microsatellites, which were used in the rest of samples. Loci were amplified using the following reactions conditions: 6.1 µl dH_2_O sterile Milli-Q (Merck Millipore), 3 µl 10x PCR buffer (20mM MgCl_2_), 1 µl (20mM MgCl_2_), 0.7 dNTPs (10mM), 0.5 µl of the unlabeled M13(-21)-tailed forward primer, 0.5 of reverse primer (10mM), 1 µl BSA (Bovine Serum Albumin), 0.1 µl Go Taq® Flexi DNA Polymerase PROMEGA (5µ/µl) and 1 µl of template DNA. Additionally, 1 µl of the fluorescently-labeled M13(-21) tail (FAM, NED, PET, VIC) (Applied Biosystems) for a final volume of 14.9 µl. PCR-amplification were performed on a T100 Thermal Cycler (Bio-Rad) using the following profiles: 95°C / 5 min, (94°C / 30 s; 57–58°C / 35 s; 72°C / 1 min) x 39 cycles; 72°C / 20 min.

PCR products were then visualized on 2.0% agarose gel and then genotyped with an ABI3730 DNA Analyzers at The University of Arizona.

### Data analyses

Alleles were visualized and sized in GeneMarker v.2.6.3 with GeneScan™ 500 LIZ (Applied Biosystems). Finally, the presence and frequency of null alleles as well as genotyping errors were assessed with Micro-Checker v.2.2.3 with a size standard [18].

Basic genetic diversity indices, number of alleles, observed (H_O_) and expected (H_E_) heterozygosities, polymorphic index content (PIC), and linkage disequilibrium (LD) tests were calculated with MStools [19]. Hardy–Weinberg equilibrium (H_WE_) by locus was assessed using a probability test with a level of significance determined by the following Markov chain parameters: 1000 dememorization steps, 100 batches and 1000 iterations per batch were analyzed using GENEPOP web v 4.2 [20], using the Weir and Cockerham estimate [21]. The Benjamini and Hochberg false discovery rate (FDR) procedure was applied to correct for multiple testing [22].

## Results and discussion

We obtained a total of 79,223,420 good quality reads (Q = 32) from the sequencing experiment, which were assembled into 441,241 contigs with an average length of 140 bp. Thirty-six microsatellite loci met the criteria and were tested (25 dinucleotide, 3 trinucleotide and 8 tetranucleotide). Of the 36 candidate loci, 16 were successfully amplified, but 4 were monomorphic and 12 consistently polymorphic. (Table 1).

**Table 1:** Microsatellite markers isolated from *Mycale cecilia*

Locus (GenBank accession number)	Motif	Primer sequence (5’->3’)		Ta	Size range (bp)	La Paz (*n* = 15)						Isabel Island (*n* = 15)					
						*PIC*	*N*_*A*_	*H*_*o*_	*H*_*E*_	*P*_*HWE*_	*NullFreq*	*PIC*	*N*_*A*_	*H*_*o*_	*H*_*E*_	*P*_*HWE*_	*NullFreq*
MYC1-15739 (OP893509)	GTTT	F:	ATAGCTACTAGGCCACCACG	57	164–261	0.797	2	0.800	0.786	0.006*	-0.0147	0.554	3	0.857	0.659	0.9280	-0.2226
		R:	AGTGACTGGGAGTGTGAGAAC														
MYC1-42219 (OP893514)	CA	F:	GTCCACTCGCAACACTTCAG	58	109–123	0.679	6	0.933	0.678	0.2639	-0.2553	0.705	4	0.800	0.722	0.0719	-0.0807
		R:	GCCAGTGTGAGTCATGTTGG														
MYC1-146780 (OP893517)	GT	F:	TTGCAAGGTGTGAGTTGAGC	58	122–164	0.871	2	0.571	0.889	0.0027*	0.1275	0.759	2	0.933	0.738	0.1402	-0.1655
		R:	GCACTTGACCACCAACTGAC														
MYC1-304443 (OP893518)	GT	F:	CAGATGTGACGTTACGGTGC	57	119–162	0.824	3	0.600	0.901	0.0034*	0.1532	0.682	7	0.867	0.701	0.4809	-0.1641
		R:	ACAAGCCCACACCTTACTTC														
MYC1-305141 (OP893519)	AC	F:	GTCTGAACCTCCATGCCATG	58	108–142	0.857	5	0.733	0.857	0.0042*	0.0601	0.589	5	0.800	0.660	0.2078	-0.1589
		R:	CGTCGTTGGCATTGAAGGG														
MYC1-22721 (OP893512)	TG	F:	CAGCGATCTCCAGGTCAAAC	58	120–148	0.733	4	0.667	0.828	0.0006*	0.0784	0.668	3	0.867	0.772	0.4430	-0.14
		R:	TCACTAGCTCCTTGTCTGCC														
MYC1-31704 (OP893513)	AC	F:	CACAGTGTACATGGTGGCAC	58	124–164	0.805	2	0.714	0.857	0.0544	0.0631	0.741	2	0.800	0.809	0.6397	-0.006
		R:	TGTTGAGTGTAAGTGTTCCTGC														
MYC1-3453 (OP893508)	ACC	F:	ACTCCCAATCACAATCACCTC	58	115–200	0.604	3	0.333	0.701	0.0012*	0.1202	0.703	4	0.733	0.800	0.1072	-0.0784
		R:	GAAATCCACCTGTGCATGGG														
MYC1-16366 (OP893510)	GT	F:	CAAGTACATGTGGCTGCACG	58	113–152	0.703	3	0.385	0.877	0.001*	0.0442	0.837	3	0.733	0.818	0.5638	-0.0116
		R:	ACCATAGCCACAATACACGC														
MYC1-109286 (OP893516)	CA	F:	GCACAAGGCGGTAAACTCTC	58	120–173	0.78	9	0.733	0.800	0.001*	0.0445	0.859	2	1.000	0.883	0.4181	0.0714
		R:	GCTGACCTACCATGATGTGC														
MYC2-19973 (OP893511)	AAC	F:	TTACATAGGTTCGCTCGCCC	58	159–211	0.658	4	0.667	0.717	0.1519	0.0019	0.692	8	0.862	0.754	0.2554	-0.009
		R:	AGGAGTCAGGGCTATTCAGG														
MYC1-95562 (OP893515)	TC	F:	GTTCAGACGTGTGCTCTTCC	58	142–170	0.566	8	0.786	0.616	0.8494	-0.2114	0.731	4	1.000	0.749	0.001*	-0.2364
		R:	GGTTCACCTGGAGAGCTTTAG														

*Ta* annealing temperature (°C), *N*_*A*_ number of alleles, *PIC* polymorphic information content, *H*_*O*_ observed heterozygosity, *H*_*E*_ expected heterozygosity, *P*_*HWE*_ Hardy-Weinberg equilibrium test and *NullFreq* Null allele frequency. Significant *p* values after False Discovery Rate method (*P < 0.05*) are shown with asterisk

Micro-Checker did not detect evidence of scoring errors. High (> 10%) null allele frequencies were found in La Paz for loci MYC1-146780, MYC1-304443 and MYC1-3453. Also, no significant values were found in the linkage disequilibrium test for any pair of loci after FDR correction (α = 0.05). All microsatellite markers were polymorphic with PIC values higher than 0.554 (range 0.566–0.871 for La Paz and 0.554–0.859 for Isabel Island), making them a helpful tool for future studies of genetic structure for *M. cecilia*. A total of 51 alleles were found in La Paz and 47 in Isabel Island, which ranged from 2 to 9 alleles per locus with a mean number of 4.25 for La Paz and 3.91 for Isabel Island.

Expected heterozygosity (HE) was overall high, with values ranging from 0.616 to 0.901. Results from GENEPOP suggested that a total of eight loci deviated from Hardy-Weinberg equilibrium (HWE) from La Paz and one from Isabel Island (Table 1), this could be due to factors unrelated to the presence of null alleles [23]. On the other hand, the heterozygotes deficiency found in nine loci in La Paz seems to be linked to HWE deviations, as seen in other sponges [24, 25]. Also, it has been suggested that it may be due to the mixing of genetic populations because of the introduction of different sources, known as the Wahlund effect [13].

*M. cecilia* is a suitable model sponge species for population genetic studies due to its abundance, widespread distribution (along the Eastern Tropical Pacific coast), and the variety of ecosystems it inhabits. Further studies using these molecular markers will help investigate the structure and gene flow among populations of this species. This is relevant in ecosystems in need of a better ecological understanding such as coral reefs, where genetic connectivity studies could significantly contribute to determining natural areas that require protection.

Cross-amplification with eight specimens of the congeneric species *Mycale microsigmatosa* was successful for 11 loci (except MYC1-19973). Therefore, they are also now being tested in additional specimens from the Gulf of Mexico and the Mexican Caribbean for population studies. Should the loci prove as polymorphic as in *M. cecilia*, these results are encouraging for the extended application of these novel microsatellite loci in other congeneric species such as *Mycale phyllophila* from the Indo-Pacific.

## Data Availability

Information on the microsatellite sequences is available in GenBank; see accession numbers in Table 1.
